# Mitral valve reoperation under ventricular fibrillation through right mini-thoracotomy using three-dimensional videoscope

**DOI:** 10.1186/1749-8090-8-81

**Published:** 2013-04-12

**Authors:** Arudo Hiraoka, Masahiko Kuinose, Toshinori Totsugawa, Genta Chikazawa, Hidenori Yoshitaka

**Affiliations:** 1 Department of Cardiovascular Surgery, The Sakakibara Heart Institute of Okayama, 2-5-1 Nakaicho, Okayama, Kita-ku, 700-0804, Japan

## Abstract

**Background:**

Conventional reoperative mitral valve surgery by median sternotomy has several difficulties. We performed mitral valve replacement (MVR) under ventricular fibrillation (VF) through right mini-thoracotomy with three-dimensional videoscope for avoiding the problems.

**Methods:**

Between 2006 and 2011, we performed 257 cases of MVR, in which 125 cases underwent isolated MVR. Ten cases of patients underwent reoperative MVR under VF through thoracotomy with three-dimensional videoscope (Group I), and 27 cases of patients underwent reoperative conventional MVR through median sternotomy (Group II). We retrospectively reviewed the outcomes and compared Group I with Group II. Preoperative left ventricular ejection fraction (LVEF) was significantly lower (50.5 ± 19.8% vs 64.4 ± 12.0%; p = 0.046), and significantly higher Euro SCORE was found in Group I (4.8 ± 2.0 vs 3.8 ± 2.4; p = 0.037).

**Results:**

Although Group I required cooling and rewarming time, average operative times was significantly shorter in Group I (262 ± 46 min vs 300 ± 57 min; p = 0.044), and cardiopulmonary bypass times and average VF times in Group I and aortic cross-clamp times in Group II were equivalent. There was no significant difference in the average of postoperative maximum creatine kinase (CK)-MB. In-hospital mortality was 0/10 (0%) and 1/27 (3.7%), and postoperative paravalvular leakage occurred in 0/10 (0%) and 1/27 (3.7%), and stroke occurred in 1/10 (10%) and 1/27 (3.7%) for Groups I and II. Two patients underwent reoperation for bleeding in Group II. Intensive care unit stay in Group I was significantly shorter than in Group II (1.8 ± 0.6 days vs 3.0 ± 1.7 days; p = 0.025).

**Conclusions:**

The higher risk of preoperative background in Group I had no effect on the operation. Mitral valve surgery under VF through right mini-thoracotomy can be an alternative procedure for reoperation after conventional various cardiothoracic surgeries.

## Background

Conventional reoperative mitral valve replacement (MVR) has several challenges and difficulties in the dissection of broad adhesions to the apex, prevention of injury of the bypassed graft and right ventricle, aortic clamp and myocardial protection. The right thoracotomy approach under ventricular fibrillation (VF) can avoid all these problems [1-3]. Reoperative mitral valve surgery through right thoracotomy has been demonstrated to be safe with similar results to sternotomy [4-6]. We introduced twin-lens three-dimensional videoscope (Shinko Optical CoLtd, Tokyo, Japan) into this procedure to obtain more clear view and accurate operation. Our study retrospectively analyzes an institutional experience with reoperative MVR under VF through right mini-thoracotomy using a three-dimensional videoscope (including previously reported one case) [7].

## Methods

From January 2006 through September 2011, 257 patients underwent MVR at the Sakakibara Heart Institute of Okayama. Isolated MVR (including tricuspid valve annuloplasty) was performed in 125 patients and 132 patients underwent concomitant procedures. In the isolated MVR cases, 10 cases were non-clamp reoperative MVR via right mini-thoracotomy under VF using three-dimensional videoscope, 27 cases were conventional reoperative MVR via median sternotomy under cardiac arrest, 20 cases were initial MVR via right mini-thoracotomy under cardiac arrest, 64 cases were initial conventional MVR via sternotomy under cardiac arrest and 4 cases were patients who had initial MVR via partial sternotomy under cardiac arrest. We reviewed this non-clamp reoperative MVR (Group I: n = 10), and compared the outcomes with conventional reoperative MVR through median sternotomy (Group II: n = 27). Table 1 shows the data of cases and patient characteristics. For the routine preoperative evaluation, all patients underwent chest X-ray, echocardiography, coronary angiography, bilateral carotid artery ultrasonography, computed tomography, magnetic resonance imaging in head and neck, and ankle-brachial index assessments. As a general rule, we selected the conventional reoperative MVR through seternotomy. We adopted the non-clamp reoperative MVR via right mini-thoracotomy for the patient with high-risk of median sternotomy approach, such as patients with patent bypassed graft, healed mediastinitis and broad adhesions between sternum and right ventricle or innominate vein. Additionally, as the procedure required right thoracotomy, one-lung ventilation and femoral-femoral cardiopulmonary bypass, we had needs to consider these factors.

**Table 1:** Preoperative characteristics

	**Group I (N = 10)**	**Group II (N = 27)**	**p value**
**Age (years)**	68.0 ± 15.0	62.8 ± 15.3	0.217
**Female**	5 (50.0%)	9 (33.3%)	0.454
**BSA (m**^**2**^**)**	1.54 ± 0.29	1.57 ± 0.18	0.411
**LVEF (%)**	50.5 ± 19.8	64.4 ± 12.0	0.046
**PAP (mmHg)**	37.1 ± 13.7	42.9 ± 13.9	0.255
**LVDd (mm)**	57.0 ± 8.7	48.2 ± 6.7	0.009
**LVDs (mm)**	42.3 ± 15.2	30.6 ± 5.9	0.009
**LAD (mm)**	46.5 ± 9.3	50.2 ± 11.9	0.339
**MR (0–4)**	3.9 ± 0.2	3.1 ± 1.1	0.046
**AF**	3 (30.0%)	11 (40.7%)	0.710
**NYHA (1–4)**	2.6 ± 0.7	2.2 ± 1.0	0.228
**Euro SCORE**	4.8 ± 2.0	3.8 ± 2.4	0.037
**Prior cardiac surgery**			
**MVP**	5 (50.0%)	9 (33.3%)	0.454
**CABG**	3 (30.0%)	1 (3.7%)	0.052
**MVR**	0 (0%)	5 (18.5%)	0.295
**AVR**	1 (10.0%)	3 (11.1%)	1.000
**Redo MVR**	1 (10.0%)	1 (3.7%)	0.473
**Congenital cardiac surgery**	0 (0%)	4 (14.8%)	0.557
**Complex valve surgery**	0 (0%)	3 (11.1%)	0.548
**Bentall procedure**	0 (0%)	1 (3.7%)	1.000

BSA, body surface area; LVEF, left ventricular ejection fraction; PAP, pulmonary artery.

pressure; Dd, dimension diastolic; Ds, dimension systolic; LAD, left atrial dimension; MR.

mitral regurgitation; AF, atrial fibrillation; NYHA, New York Heart Association; MVP.

mitral valve plasty; CABG, coronary artery bypass grafting; MVR, mitral valve replacement;

AVR, aortic valve replacement.

The mean age was not significantly different (Group I: 68 ± 15 years versus Group II: 62 ± 15 years; p = 0.217), and the mean body surface area was also not significantly different (Group I: 1.54 ± 0.29 m [2] versus Group II: 1.57 ± 0.18 m [2]; p = 0.411). There were significant differences in preoperative left ventricular ejection fraction (LVEF), left ventricular diastolic dimension (LVDd) and LV systolic dimension (LVDs). The LVDd and LVDs in Group I were significantly larger (57.0 ± 8.7 mm and 42.3 ± 15.2 mm in Group I versus 48.2 ± 6.7 mm and 30.6 ± 5.9 mm in Group II; p = 0.009 and = 0.009, respectively). The LVEF in Group I was significantly lower compared to Group II (Group I: 50.5 ± 19.8% versus Group II: 64.4 ± 12.0%; p = 0.046). Additionally, larger amount of preoperative mitral regurgitation was revealed in Group I (Group I: 3.9 ± 0.2 versus Group II: 3.1 ± 1.1; p = 0.046). Though the preoperative pulmonary artery pressure New York Heart Association (NYHA) classification was not significantly different, significantly higher Euro SCORE was found in Group I (Group I: 4.8 ± 2.0 versus Group II: 3.8 ± 2.4; p = 0.037).

In Group I, previous surgery included mitral valvuloplasty (MVP) in 4 patients, coronary artery bypass grafting (CABG) in 2 patients, redo MVR in one patient, aortic valve replacement (AVR) in one patient, mediastinitis after MVP in one patient, and CABG with Bland-White-Garland syndrome in one patient [7]. In Group II, previous surgery included MVP in 9 patients, MVR in 5 patients, congenital cardiac surgery in 4 patients, AVR in 3 patients, complex valve surgery 3 patients, redo MVR in one patient, CABG in one patient, and Bentall procedure in one patient.

### Statistical analysis

Continuous data were presented as mean ± standard deviation, and were analyzed using a Mann–Whitney test for independent data and a Wilcoxon signed rank test for paired data as appropriate. Categorical variables are given as a count and percentage of patients and compared using χ-square or Fisher’s exact test. A probability value of less than 0.05 was considered significant. All data were analyzed using the Statistical Analysis Systems software JMP 9.0 (SAS Institute Inc. Cary, NC, USA).

### Surgical technique

#### Reoperative MVR under VF through right mini-thoracotomy

After intubation with a double lumen endotracheal tube, transesophageal echocardiography was used for cardiac monitoring. The chest was opened through a right mini-thoracotomy (skin incision ≤ 10 cm) under one lung ventilation at the 4th anterolateral intercostal space. The endoscopic port was placed at the right 5th anterior intercostal space and used as a CO2 port. Intracardiac operation was performed under three-dimensional videoscope support. An arterial catheter was inserted from the right femoral artery, and venous cannulae were placed through the right femoral vein and the right internal jugular vein. In elderly patients, axillary artery could be used for arterial cannulation. Cardiopulmonary bypass was then instituted using a system for vacuum-assisted venous drainage. The pericardium was opened vertically, just 1cm medial to the right phrenic nerve. Left atrial venting was initiated through the right upper pulmonary vein. After cooling to 27–30°C, MVR was performed by conventional left atriotomy under VF. Intracardiac operation was performed using instruments for minimally invasive mitral surgery under video support. After a prosthetic valve was sewn into place in standard fashion, the left atriotomy was closed with opening the prosthetic valve by inserting a rubber catheter through the valve to left ventricle for de-airing. The de-airing and rewarming was completed, and cardioversion was performed. After recovery to normal sinus rhythm, cardiopulmonary bypass was terminated and the femoral cannulae were removed. A right pleural chest tube was positioned, and the incision was closed.

#### Reoperative MVR under cardiac arrest through median sternotomy

Cardiopulmonary bypass (CPB) was standing by and the chest was opened through median sternotomy. An arterial catheter was inserted from the right femoral artery, and venous cannula was placed through the right femoral vein. After another venous cannula was placed through the superior vena cava and CPB was established, we performed dissection of adhesion to apex. Cardiac arrest was achieved by using ascending aorta clamping and antegrade cardioplegia. MVR was performed by conventional fashion.

## Results

### Intraoperative variables

Average operative time was significantly shorter in Group I (262 ± 46 min versus 300 ± 57 min; p = 0.044). CPB times, average VF times in Group I and aortic cross-clamp times in Groups II were equivalent. The mean of reperfusion times after cardioversion in Group I was longer than the mean of reperfusion times after declamp in Group II (Group I: 33 ± 8 minutes versus Group II: 27 ± 8 minutes; p = 0.038). The average of intraoperative minimum body temperature were 27.7 ± 1.4°C in Group I and 33.7 ± 1.5°C in Group II. There were no significant differences in the average of postoperative maximum creatine kinase (CK), CK-MB and glutamic oxaloacetic transaminase (GOT). There was no case with requiring intra-aortic balloon pumping in both groups. The perioperative data of the two groups are shown in Table 2.

**Table 2:** Perioperative data

	**Group I (N = 10)**	**Group II (N = 27)**	**p value**
**Operative time (min)**	262 ± 46	300 ± 57	0.044
**CPB time (min)**	145 ± 25	135 ± 28	0.281
**Cross-clamp time (min)**		84 ± 19	0.120
**VF time (min)**	90 ± 7		
**Reperfusion time after declamp (min)**		27 ± 8	0.038
**after cardioversion (min)**	33 ± 8		
**Minimum BT (°C)**	27.7 ± 1.4	33.7 ± 1.5	< 0.0001
**Inotropic agent support**	2 (20.0%)	6 (22.2%)	1.000
**Maximum CK**	740.8 ± 436.4	591.5 ± 452.3	0.266
**CK-MB (IU/L)**	35.0 ± 10.7	60.5 ± 64.9	0.155
**Maximum GOT (IU/L)**	77.0 ± 17.5	80.7 ± 50.3	0.320

CPB, cardiopulmonary bypass; VF, ventricular fibrillation; BT, body temperature; CK, creatine.

kinase; GOT, glutamic oxaloacetic transaminase.

### Postoperative variables

In-hospital mortality was 0/10 (0%) and 1/27 (3.7%) for Groups I and II. Cause of death in Group II was sepsis. Postoperative paravalvular leakage and late cardiac tamponade occurred in 0/10 (0%) and 1/27 (3.7%), and stroke occurred in 1/10 (10%) and 1/27 (3.7%) for Groups I and II. The patient with stroke in Group I had cerebellar hemorrhage requiring emergent craniotomy at 6 days after operation. After the operation, the patient recovered well. Although 2 patients underwent reoperation for bleeding in Group II, there was no reoperation for bleeding in Group I. New onset atrial fibrillation occurred in 0/10 (0%) and 3/27 (11.1%) for Groups I and II. There was no significant difference of average times of postoperative ventilation, and postoperative hospital stay (7.6 ± 4.2 hours in Group I versus 17.8 ± 33.7 hours in Group II; p = 0.700 and 18.3 ± 8.4 days in Group I versus 21.5 ± 13.2 days in Group II; p = 0.537). Intensive care unit (ICU) stay in Group I was significantly shorter than Group II (1.8 ± 0.6 days in Group I, 3.0 ± 1.7 days in Group II; p = 0.025). Although postoperative LVEF was lower in Group I (Group I: 46.5 ± 18.6% versus Group II: 64.3 ± 8.8%; p = 0.004), postoperative NYHA classification was equivalent (Group I: 1.3 ± 0.5% versus Group II: 1.3 ± 0.7%; p = 0.840). There were no significant differences in improvement rate of cardiac function. Table 3 compares the two groups regarding postoperative variables.

**Table 3:** Postoperative data

	**Group I (N = 10)**	**Group II (N = 27)**	**p value**
**Postoperative ventilation time (hours)**	7.6 ± 4.2	17.8 ± 33.7	0.700
**ICU stay (days)**	1.8 ± 0.6	3.0 ± 1.7	0.025
**Postoperative hospital stay (days)**	18.3 ± 8.4	21.5 ± 13.2	0.537
**LVEF (%)**	46.5 ± 18.6	64.3 ± 8.8	0.004
**LVDd (mm)**	51.0 ± 14.2	46.2 ± 5.5	0.545
**LVDs (mm)**	38.9 ± 15.3	29.8 ± 5.0	0.050
**Change rate of LVEF (%)**	−8.7 ± 28.1	3.7 ± 18.0	0.184
**Change rate of LVDd (mm)**	−11.8 ± 12.1	−3.4 ± 11.0	0.067
**Change rate of LVDs (mm)**	−8.7 ± 12.3	−2.2 ± 13.3	0.198
**NYHA (1–4)**	1.3 ± 0.5	1.3 ± 0.7	0.840
**Complications**			
**Death**	0 (0%)	1 (3.7%)	1.000
**Paravalvular leakage**	0 (0%)	1 (3.7%)	1.000
**Stroke**	1 (10.0%)	1 (3.7%)	0.473
**Late cardiac tamponade**	0 (0%)	1 (3.7%)	1.000
**Reoperation for bleeding**	0 (0%)	2 (7.4%)	1.000
**New onset AF**	0 (0%)	3 (11.1%)	0.548

ICU, intensive care unit; LVEF, left ventricular ejection fraction; NYHA, New York Heart.

Association; AF, atrial fibrillation.

## Discussion

Conventional reoperative MVR has several challenges and difficulties. In a detailed review of the STS database, it was demonstrated that the risk of mortality for isolated MVR increased from 5.09% to 9.25% in the presence of a previous cardiac operation [8]. These findings were confirmed in a more recent review of the same database that demonstrated that reoperation was associated with significantly increased mortality for all valve operations [9]. Division of the sternum carries an increased risk of injury of major cardiac structures in the presence of adhesions with the right ventricle, innominate vein and bypassed grafts [10]. Thus, the injury can be a cause of surgical mortality. Additionally, the conventional approach requires dissection of broad adhesions to the apex, aortic clamp and myocardial protection. In the case that requires mitral valve surgery after deep sternal wound infection, we have no other choice to avoid conventional approach through median sternotomy [11].

Recently, minimally invasive cardiac surgery (MICS) for mitral valve surgery has been developed. MICS was associated with a mortality rate similar to that for open sternotomy cases, reduced length of intensive care unit and hospital stays, fewer blood transfusions, and a faster return to normality compared with conventional operative approaches [12,13]. Similarly, Yamada and colleagues reported that MICS was associated with earlier recovery of daily activities and improved quality of life in the early perioperative period [14]. Right mini-thoracotomy approach for mitral valve has been established and applied to mitral valve reoperation [1,3-5]. Port-access technique with thoracoscopic support for initial and reoperative mitral valve surgery is also safe and effective [5,15-17]. We introduced mini-thorocotomy approach into mitral valve surgery from 2005, and 156 cases of minimally invasive mitral valve surgery were performed. We also applied this technique to reoperative mitral valve surgery and selected non-clamp procedure under VF and mild hypothermia. Right mini-thoracotomy approach can achieve excellent operative view for the mitral valve without dissection of adhesions to the apex. Additionally, we used three-dimensional videoscope to optimally visualize the mitral valve, as the efficiency has been reported [18,19]. The procedure under VF does not require dissection of adhesions around the ascending aorta and can avoid several difficulties regarding aortic clamp and myocardial protection. As compared to mitral valve surgery with cardioplegic arrest, the efficiency and safety of this combination have been reported [6]. Our combination of right mini-thoracotomy, non-clamp under VF and three-dimensional videoscope support for reoperative mitral valve surgery suggests the similar conclusion. Although intraoperative myocardial protection is a major concern, the safety of mitral valve surgery under VF through right thoracotomy in the cases after CABG with functioning internal mammary artery grafts has been reported [2,20].

In our study, although Group I required cooling and rewarming time, average CPB times were equivalent to Group II. The operation time in Group I was shorter than Group II, and the process of operation was very smooth in Group I. The average of postoperative maximum CK, CK-MB and GOT were not significantly different, and the lower preoperative EF and larger amount of preoperative mitral regurgitation in Group I had no effect on the operation. These data demonstrated intraoperative myocardial protection under mild hypothermia and VF was not an inappropriate myocardial protection strategy compared to cardiac arrest with cardioplegic solution. As there was no hospital mortality, postoperative paravalvular leakage, and reoperation for bleeding in Group I, the risk of complications was lower compared to Group II. The stroke occurred in Group I was not due to thromboembolism or arterial cannulation, but small cerebral aneurysm. Although there was no significant difference of average times of postoperative ventilation, and postoperative hospital stay, ICU stay in Group I was significantly shorter than Group II. Moreover, although preoperative Euro SCORE in Group I was significantly higher, postoperative course in Group I were thought to be satisfactory compared to Group II. These results suggest that reoperative MVR under VF through right mini-thoracotomy is a reasonable alternative to redo sternotomy for patients with previous sternotomy.

This study has several limitations. First of all, this study was not a randomized study, and the number of patients of cohort was small to prove the efficacy of this procedure. Secondly, pathological data of myocardium was not obtained and we cannot evaluate the damage of myocardium histogenetically. Finally, these procedures were performed by two different surgeons, and the bias cannot be denied.

## Conclusions

This early clinical experience suggests that the outcomes of reoperative MVR under VF through right mini-thoracotomy were acceptable as one of effective options for patients who required reoperative mitral valve surgery.

## Abbreviations

MVR: Mitral valve replacement; VF: Ventricular fibrillation; LVEF: Left ventricular ejection fraction; LVDd: Left ventricular diastolic dimension; LVDs: Left ventricular systolic dimension; NYHA: New York Heart Association; MVP: Mitral valvuloplasty; CABG: Coronary artery bypass grafting; AVR: Aortic valve replacement; CPB: Cardiopulmonary bypass; CK: Creatine kinase; GOT: Glutamic oxaloacetic transaminase; ICU: Intensive care unit.

## Competing interests

The authors declare that they have no competing interests.

## Authors’ contributions

AH and MK acted in conception and design. AH acted in data analysis and interpretation. AH and GC acted in manuscript writing. MK, GC, TT, and HY acted in revision of the article. All authors read and approve the final manuscript.
